# Supplementary material for: Implication of Central Nervous System Barrier Impairment in Amyotrophic Lateral Sclerosis: Gender-Related Difference in Patients
Source: Int J Mol Sci. 2023 Jul 7;24(13):11196. doi: 10.3390/ijms241311196 (PMC10342931; doi:10.3390/ijms241311196)
Supplement: Supplementary file 1 [file ijms-24-11196-s001.zip › ijms-2474952-supplementary.pdf]

Table S1: clinical characteristic of included patients in the genetic analysis

	**All patients (N=171)**
Onset age (year)	67 (61.00, 72.50)
Onset site	
Spinal (N,%)	119 (69.6)
Bulbar (N,%)	50 (29.2)
Respiratory (N,%)	2 (1.2%)
Diagnostic delay (months)	8.99 (5.03,14.00)
At diagnosis	
FVC (%)	95.3 (76.6, 111.2)
Weight (kg)	69.0 (61.0, 77.8)
ALSFRS-r	40.4 (36.4, 43.5)
dALSFRS	
Variation in reference weight	-2.47 (-7.96, 0.00)
Variation after a year (%)	
FVC	-18.2 (-32.9, -10.2)
Weight	-0.90 (-6.5, 2.6)
ALSFRS	-21.7 (-41.5, -12.3)
Disease duration before death (months)	25.7 (17.7, 32.1)

Continuous variables are represented as median (Interquartile range.). Information about diagnostic dela ,ALSFRS-r and FVC at diagnosis, variation of ALSFRS, FVC and weight over a year, and about death was missing for 5.3, 0.6, 21.1, 42.7, 79.7, 43.9 and 45.6 % of patients, respectively. ALSFRS-r: ALS functional rating scale-revised ; dALSFRS-r : Progression rate of ALSFRS-R; FVC : Forced Vital Capacity.
